# Surgical Anatomy

**Published:** 1850-12

**Authors:** 


Art. X.—*Surgical Anatomy.* By Joseph Maclise, Surgeon.—With colored plates. Part third. Philadelphia: Lea & Blanchard. 1850.

This part amply sustains the reputation acquired by the preceding parts, We think this 'Surgical Anatomy'

by far the best we have seen. The plates in part third are devoted to the various forms of Hernia, and we could scarcely say more in praise of them, than in saying they are superior to Sir Astley Cooper's. We doubt, indeed, whether there is any work in the language, on the subject of hernia, that makes any approach to the excellence of the plates, and the accompanying commentaries.

We shall not enter into details of the contents of Maclise's "Surgical Anatomy," for we take it for granted that physicians will examine it, either in commencing a library, or in keeping one up. We regard it as indispensable to the office of a physician, and the publishers deserve the thanks of the profession for the very admiriable manner in which they have issued the work. The plates are highly creditable to American art.

